# Lorlatinib with or without chemotherapy in ALK-driven refractory/relapsed neuroblastoma: phase 1 trial results

**DOI:** 10.1038/s41591-023-02297-5

**Published:** 2023-04-03

**Authors:** Kelly C. Goldsmith, Julie R. Park, Kimberly Kayser, Jemily Malvar, Yueh-Yun Chi, Susan G. Groshen, Judith G. Villablanca, Kateryna Krytska, Lillian M. Lai, Patricia T. Acharya, Fariba Goodarzian, Bruce Pawel, Hiroyuki Shimada, Susan Ghazarian, Lisa States, Lynley Marshall, Louis Chesler, Meaghan Granger, Ami V. Desai, Rajen Mody, Daniel A. Morgenstern, Suzanne Shusterman, Margaret E. Macy, Navin Pinto, Gudrun Schleiermacher, Kieuhoa Vo, Holger C. Thurm, Joseph Chen, Marlon Liyanage, Gerson Peltz, Katherine K. Matthay, Esther R. Berko, John M. Maris, Araz Marachelian, Yael P. Mossé

**Affiliations:** 1 Aflac Cancer and Blood Disorders Center, Children’s Healthcare of Atlanta, Atlanta, GA USA; 2 Winship Cancer Institute, Emory University School of Medicine, Atlanta, GA USA; 3 Seattle Children’s Hospital, Seattle, WA USA; 4 Department of Pediatrics, University of Washington School of Medicine, Seattle, WA USA; 5 Department of Psychiatry and Behavioral Sciences, Vanderbilt University Medical Center, Nashville, TN USA; 6 Cancer and Blood Disease Institute, Children’s Hospital Los Angeles, Los Angeles, CA USA; 7 Keck School of Medicine, University of Southern California, Los Angeles, CA USA; 8 Division of Oncology and Center for Childhood Cancer Research, Children’s Hospital of Philadelphia, Philadelphia, PA USA; 9 Department of Radiology, University of Iowa Hospital and Clinics, Iowa City, IA USA; 10 Department of Radiology, Children’s Hospital Los Angeles, Los Angeles, CA USA; 11 Department of Pathology and Laboratory Medicine, Children’s Hospital Los Angeles, Los Angeles, CA USA; 12 Department of Pathology and Pediatrics, Stanford University School of Medicine, Stanford, CA USA; 13 Perelman School of Medicine at the University of Pennsylvania, Philadelphia, PA USA; 14 The Royal Marsden Hospital, London, UK; 15 The Institute of Cancer Research, London, UK; 16 Cook Children’s Medical Center, Fort Worth, TX USA; 17 Department of Pediatrics, Section of Hematology/Oncology/Stem Cell Transplantation, University of Chicago, Chicago, IL USA; 18 Department of Pediatrics, University of Michigan, Ann Arbor, MI USA; 19 Division of Haematology and Oncology, Hospital for Sick Children, Toronto, ON Canada; 20 Department of Paediatrics, University of Toronto, Toronto, ON Canada; 21 Dana-Farber Cancer Institute, Boston Children’s Cancer and Blood Disorders Center, Harvard Medical School, Boston, MA USA; 22 University of Colorado Anschutz Medical Campus, Aurora, CO USA; 23 Center for Cancer and Blood Disorders, Children’s Hospital Colorado, Aurora, CO USA; 24 RTOP (Recherche Translationelle en Oncologie Pédiatrique), INSERM U830, Research Center, PSL Research University, Institut Curie, Paris, France; 25 SIREDO Oncology Center (Care, Innovation and Research for Children, Adolescents and Young Adults with Cancer), Institut Curie, Paris, France; 26 Department of Pediatrics, UCSF Benioff Children’s Hospital, University of California, San Francisco School of Medicine, San Francisco, CA USA; 27 Global Product Development, Clinical Pharmacology, Pfizer Oncology, Pfizer, Inc., New York, NY USA; 28 Division of Pediatric Hematology and Oncology, Schneider Children’s Medical Center, Petach Tikva, Israel; 29 Faculty of Medicine, Tel Aviv University, Tel Aviv, Israel

**Keywords:** Paediatric cancer, Drug development

## Abstract

Neuroblastomas harbor *ALK* aberrations clinically resistant to crizotinib yet sensitive pre-clinically to the third-generation ALK inhibitor lorlatinib. We conducted a first-in-child study evaluating lorlatinib with and without chemotherapy in children and adults with relapsed or refractory ALK-driven neuroblastoma. The trial is ongoing, and we report here on three cohorts that have met pre-specified primary endpoints: lorlatinib as a single agent in children (12 months to <18 years); lorlatinib as a single agent in adults (≥18 years); and lorlatinib in combination with topotecan/cyclophosphamide in children (<18 years). Primary endpoints were safety, pharmacokinetics and recommended phase 2 dose (RP2D). Secondary endpoints were response rate and ^123^I-metaiodobenzylguanidine (MIBG) response. Lorlatinib was evaluated at 45–115 mg/m^2^/dose in children and 100–150 mg in adults. Common adverse events (AEs) were hypertriglyceridemia (90%), hypercholesterolemia (79%) and weight gain (87%). Neurobehavioral AEs occurred mainly in adults and resolved with dose hold/reduction. The RP2D of lorlatinib with and without chemotherapy in children was 115 mg/m^2^. The single-agent adult RP2D was 150 mg. The single-agent response rate (complete/partial/minor) for <18 years was 30%; for ≥18 years, 67%; and for chemotherapy combination in <18 years, 63%; and 13 of 27 (48%) responders achieved MIBG complete responses, supporting lorlatinib’s rapid translation into active phase 3 trials for patients with newly diagnosed high-risk, ALK-driven neuroblastoma. ClinicalTrials.gov registration: NCT03107988.

## Abstract (web summary)

In children, adolescents and adults with mutated ALK-driven relapsed or refractory neuroblastoma, a third-generation ALK inhibitor with or without chemotherapy was well tolerated, and recommended phase 2 doses were successfully identified in all patient groups.

## Main

Neuroblastoma is an aggressive extracranial solid tumor of childhood that is responsible for a disproportionate number of pediatric cancer-related deaths^1^. Gain-of-function mutations in the *ALK* (anaplastic lymphoma kinase) oncogene were identified as the genetic etiology of familial neuroblastoma and as the most common somatic single-nucleotide variants in neuroblastoma, positioning *ALK* as the most frequently mutated oncogene tractable for targeted therapy in neuroblastoma^2–5^. Furthermore, relapsed neuroblastoma harbors increased somatic mutations, with enrichment of *ALK*-activating subclonal/clonal mutations compared to diagnostic tumors, with a frequency of 20% and rising as we sequence patient tumors and/or plasma more routinely at time of relapse^6–9^. ATP-competitive ALK/Met/ROS1 tyrosine kinase inhibitors (TKIs), such as crizotinib, have transformed the treatment of patients with non-small cell lung carcinoma (NSCLC) harboring *ALK* translocations^10^ and demonstrated differential activity in pre-clinical models of ALK-driven neuroblastoma^11^. A phase 1 trial of ceritinib in children with ALK-driven malignancies showed a response rate (RR; complete/partial) of 20% (6/30) with responses primarily observed in patients with an ALK R1275 mutation^12^. A phase 2 trial of crizotinib in children with refractory or relapsed ALK-mutant neuroblastoma reported an RR of 15% (3/20);^13^ in stark contrast, far more objective and sustained responses were observed in ALK-fusion-driven refractory or relapsed anaplastic large cell lymphoma (RR 90%) and inflammatory myofibroblastic tumors (RR 86%)^14^, highlighting the difference between therapeutic targeting of full-length mutated *ALK* in neuroblastoma compared to cytoplasmic ALK fusion proteins in other cancers. Primary resistance to crizotinib and early-generation ALK inhibitors represents a major obstacle for common *ALK* hotspot mutations in neuroblastoma (for example, F1174L and F1245C)^11,15–18^, supporting the need for next-generation ALK TKIs with improved selectivity and potency against neuroblastoma de novo resistant *ALK* mutations.

Lorlatinib, a third-generation macrocyclic inhibitor of ALK and ROS1, was developed to maintain potency across resistant *ALK* mutations that arise in response to first-generation or second-generation ALK TKIs in NSCLC^19^. Early-phase clinical trials in adults with ALK-driven or ROS1-driven NSCLC demonstrated objective and durable responses to single-agent lorlatinib, including patients with central nervous system (CNS) metastases and those previously treated with other ALK TKIs^20^. One dose-limiting toxicity (DLT) of grade 2 cognitive effects occurred in an adult receiving the 200 mg daily dose. The adult recommended phase 2 dose (RP2D) was determined to be 100 mg per day, two dose levels below the DLT^20^. Although common adverse events (AEs) of peripheral edema and neuropathy were similar to other ALK TKIs, unique AEs of hypertriglyceridemia, hypercholesterolemia and CNS effects were also observed^21^.

Lorlatinib demonstrates high potency across acquired *ALK-*activating mutations in adult cancers, including the intractable *ALK* variants (F1174L and F1245C) found de novo in neuroblastoma^21–24^. Lorlatinib exerts potent activity in ALK-driven neuroblastoma pre-clinical models in vivo, with anti-tumor doses 10–30-fold lower than crizotinib^25^. Lorlatinib induced complete tumor regression in both crizotinib-resistant and sensitive neuroblastoma-derived xenografts harboring F1174L, F1245C or R1275Q *ALK* mutations, demonstrating lorlatinib’s potential to overcome crizotinib resistance. These data provided the pre-clinical rationale for the clinical development of lorlatinib for patients with ALK-driven neuroblastoma.

**Fig. 1:**
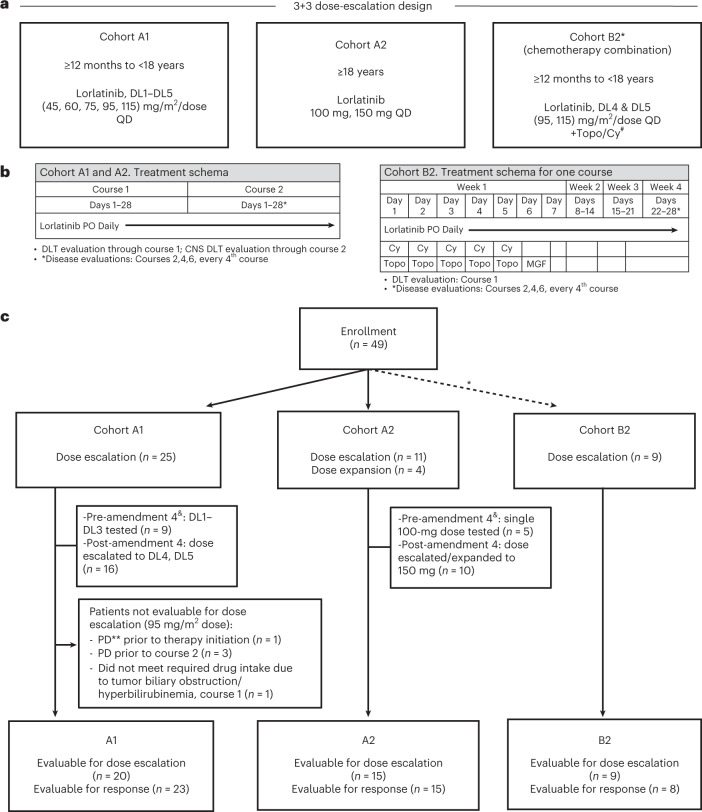
Study design and patient disposition. **a**, NANT 2015-02 study design. **b**, Treatment regimen by cohort. **c**, Patient distribution. *Cohort B2 opened at 95 mg/m^2^ once cohort A1, DL4 (95 mg/m^2^) was deemed tolerable and safe. B2 did not dose escalate to 115 mg/m^2^ until both B2, DL4 (95 mg/m^2^) in chemotherapy combination and cohort A1, DL5 (115 mg/m^2^) as single agent were deemed tolerable and safe. ^&^Amendment 4: Additional doses of lorlatinib were added to dose-escalation cohorts A1 and A2 based on safety/PK data at the lower doses achieved, and pre-clinical data showing lorlatinib doses required for anti-neuroblastoma potency were higher than NSCLC pre-clinical models. ^#^Topo/Cy, topotecan/cyclophosphamide; MGF, myeloid growth factor; **PD, progressive disease. PO, orally; QD, once a day.

Here we present the safety, tolerability and anti-tumor activity of a first-in-child New Approaches to Neuroblastoma Therapy (NANT) Consortium phase 1 study (NANT2015-02) of lorlatinib in children, adolescents and adults with ALK-driven refractory or relapsed neuroblastoma. Primary aims were to determine the toxicities, pharmacokinetics and RP2D of lorlatinib administered both as monotherapy and in combination with topotecan/cyclophosphamide. A secondary aim was to evaluate the anti-tumor activity by determining the RR (complete response (CR) and partial response (PR)) and a modified RR (CR/PR/minor responses (MRs)) (as defined in NANT version 2.0 response criteria^26^). This trial had four parts (Fig. 1a): phase 1 dose escalation for <18 years (A1); phase 1 dose escalation for ≥18 years (A2); dose escalation for lorlatinib with topotecan/cyclophosphamide for <18 years (B2), all determined by 3 + 3 design; and dose expansion for single-agent lorlatinib (B1). We report the data for cohorts A1, A2 and B2 that have met the pre-specified protocol endpoints for determination of the RP2D, description of toxicity and activity. Dose levels (DLs) and treatment regimens for each cohort are described in Fig. 1a,b. Patients ≥12 months with measurable or evaluable refractory or relapsed high-risk neuroblastoma, including CNS metastases and/or prior treatment with ALK TKIs aside from lorlatinib, were eligible (Methods). DLTs occurring in course 1 and DLTs from CNS effects (CNS DLTs) occurring in courses 1 and 2 informed dose escalation and RP2D determination. Patients were evaluable for dose escalation if they had received ≥75% of expected doses or experienced a DLT in courses 1 or 2. Neurobehavioral functioning was routinely assessed to provide real-time measures of cognition, behavior and mood during lorlatinib administration. Patients who received any amount of lorlatinib were considered evaluable for response unless deemed by central review to have inadequate imaging to assess overall response. An exploratory aim to prospectively determine the frequency of circulating tumor cell-free DNA (ctDNA) detection of *ALK* and acquired mutations at study entry and with each disease evaluation was performed and will be reported separately (Berko, *et al.*, Nat. Comm. 2023, in press).

## Results

### Participants

Between September 2017 and February 2022, 49 patients were enrolled (A1 *n* = 25, 9/5/2017–2/10/2021; A2 *n* = 15, 11/1/2017–7/27/2021; and B2 *n* = 9, 2/10/2020–12/3/2021; Fig. 1c and Table 1). Of 34 patients <18 years of age (A1 and B2), 11 (33%) had *MYCN-*amplified tumors compared to one (7%) patient ≥18 years of age (A2). Nine patients (18%) had refractory neuroblastoma, and the remainder had relapsed disease (*n* = 40, 82%), and no patients had CNS parenchymal metastases. Patients were heavily pre-treated with a median time from diagnosis to study enrollment of 23.5 months (range 7.5–253 months), including patients who had previously received myeloablative chemotherapy followed by autologous stem cell transplant (59%) and/or GD2-directed immunotherapy (71%), and 41% who had received a prior ALK inhibitor, with specific details in Table 1. Patients previously treated with an ALK TKI had a median of 8.4 months (range 0.2–41.6 months) of prior TKI therapy. Five patients (all cohort A1) were not evaluable for dose escalation (see Fig. 1c for reasons), and three patients (two cohort A1 and one cohort B2) were not evaluable for response.

**Table 1:** Baseline patient characteristics

Characteristic	Overall, *n* = 49	Cohort A1, *n* = 25	Cohort A2, *n* = 15	Cohort B2, *n* = 9
**Gender, no. (%)**				
Male	24 (49%)	12 (48%)	6 (40%)	6 (67%)
Female	25 (51%)	13 (52%)	9 (60%)	3 (33%)
**Years of age at study entry**				
Median (range)	9.08 (2.51, 50.45)	6.28 (2.51, 17.18)	24.83 (15.24, 50.45)	6.67 (3.68, 12.69)
**Time (months) from diagnosis to study enrollment**				
Median time (range)	23.46 (7.46, 253.42)	24.47 (8.38, 179.4)	31.11 (11.50, 253.4)	16.95 (7.46, 43.89)
**Race, no. (%)**				
White	37 (76%)	18 (72%)	13 (87%)	6 (67%)
Black	2 (4%)	2 (8%)	0 (0%)	0 (0%)
Other	1 (2%)	1 (4%)	0 (0%)	0 (0%)
Not specified	9 (18%)	4 (16%)	2 (13%)	3 (33%)
**Disease status, no. (%)**				
Relapsed	40 (82%)	22 (88%)	12 (80%)	6 (67%)
Refractory/persistent	9 (18%)	3 (12%)	3 (20%)	3 (33%)
***MYCN status at diagnosis, no. (%)***				
Amplified	12 (24%)	8 (32%)	1 (7%)	3 (33%)
Not amplified	37 (76%)	17 (68%)	14 (93%)	6 (67%)
**Prior therapy received, no. (%)**				
Myeloablative transplant (yes)	29 (59%)	16 (64%)	7 (47%)	6 (67%)
GD2 immunotherapy (yes)	35 (71%)	20 (80%)	9 (60%)	6 (67%)
Topotecan/cyclophosphamide (yes)	34 (69%)	19 (76%)	9 (60%)	6 (67%)
Disease status at baseline, no. (%)				
Soft tissue target lesion(s) present	35 (71%)	23 (92%)	8 (53%)	4 (44%)
Bone marrow involved	17 (35%)	8 (32%)	5 (33%)	4 (44%)
Bone involved	38 (78%)	17 (68%)	13 (87%)	8 (89%)
***ALK*** **aberration, no. (%)**				
F1174 (C or L)	25 (51%)	12 (48%)	8 (53%)	5 (56%)
F1245 (Y or L)	6 (12%)	6 (24%)	0 (0%)	0 (0%)
R1275 (Q or L)	16 (33%)	6 (24%)	6 (40%)	4 (44%)
D1276_R1279>E	1 (2%)	0 (0%)	1 (7%)	0 (0%)
ALK amplification	1 (2%)	1 (4%)	0 (0%)	0 (0%)
**Prior ALK inhibitor therapy, no. (%)**				
ALK inhibitor naive	29 (59%)	16 (64%)	10 (67%)	3 (34%)
Crizotinib	3 (6%)	2 (8%)	1 (7%)	0 (0%)
Other ALK inhibitor^*^	1 (2%)	0 (0%)	1 (7%)	0 (0%)
Crizotinib + chemotherapy^§^	9 (18%)	4 (16%)	2 (13%)	3 (33%)
Other ALK inhibitor + chemotherapy	4 (8%)	1 (4%)	0 (0%)	3 (33%)
More than one ALK inhibitor	3 (6%)	2 (8%)	1 (7%)	0 (0%)
Duration of ALK TKI therapy, range (months)	8.39 (0.20, 41.60)	8.51 (2.66, 19.68)	20.11 (6.64, 41.59)	1.30 (0.20, 11.04)
**Tumor tissue used for ALK testing, no. (%)**				
Diagnosis	9 (18%)	4 (16%)	2 (13%)	3 (33%)
Second look surgery	5 (10%)	2 (8%)	2 (13%)	1 (11%)
Relapsed/progression	31 (63%)	16 (64%)	10 (67%)	5 (56%)
Persistent/refractory	4 (8%)	3 (12%)	1 (7%)	0 (0%)

§ Two patients had crizotinib alone and also with chemotherapy.

*Ceritinib, alectinib or ensartinib

All common neuroblastoma *ALK* hotspot mutations were represented in tumor sequencing results for eligibility, including F1174C/L (51%), R1275Q (33%) and F1245Y/L (12%), one *ALK* amplification and one pathologic D1276_R1279 *ALK*-activating mutation (Table 1). Tumor tissue for CLIA-certified sequencing of *ALK* came from diagnostic tumor or primary tumor resections in ten (25%) relapsed and five (55%) refractory patients and from biopsies at time of relapsedrefractory disease for the remainder of patients. The *ALK* variant allele frequency (VAF) was available for 33 of the enrollment tumor sequencing results and ranged from 2% to 57%, with nine having *ALK* VAF < 20%. In the 37 patients with detectable baseline ctDNA, using the FoundationOne Liquid CDx^27^, *ALK* mutations detected in patient plasma at study entry were identical to the alterations seen in the tumor tissue used for study eligibility. Three patients had additional *ALK* alterations detected by ctDNA at baseline along with the identified tumor *ALK* alteration (Supplementary Table 1), two of whom had received a prior ALK TKI. Further ctDNA results are described separately (Berko, *et al.*, Nat. Comm, in press).

### Safety and RP2D

A summary of DLTs used to determine dose escalation (all DLTs in course 1 plus CNS DLTs through course 2) and delayed DLTs are presented in Table 2. In cohort A1, a single DLT (grade 3 diarrhea) occurred at DL5 in a patient with baseline lactose intolerance. Notably, lorlatinib tablets contain lactose monohydrate as an excipient. The AE resolved with oral administration of lactase enzyme, and the patient subsequently tolerated reduced-dose lorlatinib (DL4) for 11 total courses. An additional patient in cohort A1 (DL5) developed delayed DLT (course 3) of grade 2 insomnia and hallucinations requiring drug interruption but tolerated rechallenge at DL4, receiving 14 total courses (Extended Data Table 1). The RP2D for single-agent lorlatinib in patients <18 years of age was determined to be 115 mg/m^2^/dose daily (DL5), approximately 200% of the adult RP2D^21^.

**Table 2:** DLTs by cohort and DL

Cohort	DL	No. of patients entered and eligible	Evaluable for dose-escalation decisions	Patients with DLT in course 1*	Patients with DLT in course 2*	Patients with DLT in other courses
A1	DL1 (45 mg/m^2^)	3	3	0	0	0
	DL2 (60 mg/m^2^)	3	3	0	0	0
	DL3 (75 mg/m^2^)	3	3	0	0	0
	DL4 (95 mg/m^2^)	10	5^a^	0	0	0
	DL5 (115 mg/m^2^)	6	6	1^b^	0	1^b^
A2	100 mg	5	5	0	0	2^c^
	150 mg	10	10	1^d^	2^e,f^	3^e,f,g^
B2	DL4 (95 mg/m^2^)	3	3	0	0	0
	DL5 (115 mg/m^2^)	6	6	0	0	1^h^

a. Five patients were not evaluable for dose-escalation decisions (Fig. 1).

b. Grade 3 diarrhea in course 1 in one patient; grade 2 hallucinations/insomnia in course 3 in another patient.

c. Grade 3 hyperglycemia in course 3 in one patient; grade 2 memory impairment in course 7 in another patient.

d. Grade 2–4 agitation/anxiety/mania in course 1.

e. Grade 2–4 delusions/psychosis/suicide attempt in course 2 that recurred in course 3 despite dose reduction.

f. Grade 3 agitation/neuropathy in course 2 that recurred in course 3 despite dose reduction.

g. Grade 2 edema limbs and neuropathy in course 4.

h. Grade 2 anxiety/depression/somnolence/hypersomnia in course 3.

*DLTs used for dose escalation include all DLTs occurring in course 1 and any CNS DLT through course 2.

In cohort A2, 100 mg dose, no DLTs occurred in courses 1 and 2. One adult had lorlatinib held for grade 2 memory impairment (course 7) that met criteria for late DLT that resolved with dose hold (Extended Data Table 1). Another baseline obese adult had a dose reduction for hyperglycemia/glucose intolerance that was medically managed (course 3) and received ten courses. The study was subsequently amended to exclude medically manageable hyperglycemia without glucose intolerance, in keeping with the adult NSCLC experience^28^. In cohort A2, 150 mg dose, one patient experienced a DLT of grade 4 psychosis, hallucinations and suicidal ideation/attempt during course 2. Notably, the patient had a prior history concerning for a psychiatric disorder not disclosed before enrollment. The symptoms recurred despite dose reduction in course 3, necessitating cessation of therapy and prompting an amendment to exclude rechallenge for grade 4 neuropsychiatric toxicity. No other courses 1 and 2 DLTs occurred during the dose-escalation phase, defining 150 mg daily dosing as the single-agent RP2D for patients ≥18 years of age. In the A2, 150 mg dose expansion cohort, a DLT of grade 4 mania and grade 3 memory impairment, anxiety and delusions occurred (course 1) in a patient who was concurrently taking marijuana, which resolved with lorlatinib discontinuation; no drug rechallenge occurred. Additionally, one patient had a DLT of grade 3 agitation and neuropathy in course 2 that recurred in course 3 despite dose reduction and resolved after discontinuation of lorlatinib. One other patient had a delayed DLT in course 4 of grade 2 neuropathy/edema but has tolerated reduced-dose lorlatinib. For the entire cohort of patients ≥18 years of age treated at 150 mg (*n* = 10, dose escalation and expansion), five (50%) required dose reduction due to various toxicities, not all meeting DLT criteria (Extended Data Table 1).

In cohort B2, no DLTs were observed at DL4 or DL5 of lorlatinib in combination with fixed doses of topotecan/cyclophosphamide. A delayed DLT of grade 2 anxiety, depression, somnolence and hypersomnia occurred in a 12-year-old on DL5 requiring dose interruption but tolerated rechallenge at DL4, receiving a total of five courses. These data supported DL5, 115 mg/m^2^/dose daily, for lorlatinib in combination with topotecan/cyclophosphamide as the RP2D for patients <18 years of age.

Overall, the most common grade 1–4 treatment-related AEs for single-agent lorlatinib across all ages were hypertriglyceridemia (90%), weight gain (87%) and elevated cholesterol (79%) (Extended Data Table 2). Grade 1–2 peripheral limb edema occurred mainly in patients ≥18 years of age (47%) compared to <18 years of age (4%). Patients <18 years of age also had lower incidence of peripheral neuropathy compared to adults (13% versus 27%, respectively). The most common AEs observed after lorlatinib combined with topotecan/cyclophosphamide in patients <18 years of age were the expected hematologic toxicities of chemotherapy (Extended Data Table 3), with hypertriglyceridemia (89%) and hypercholesterolemia (78%) being the most common non-hematologic AEs. For all patients <18 years of age (cohorts A1 and B2), two of 33 (6%) developed grade 2 CNS effects with only one requiring dose reduction, with no grade 3–4 CNS effects observed (Extended Data Table 4). For patients ≥18 years of age (cohort A2), four of 15 (27%) developed grade 2–4 CNS effects, two requiring dose hold/reduction and two requiring discontinuation.

Real-time neurobehavioral screening identified 12 of 48 (25%) patients across all cohorts (A1, A2 and B2) who had baseline pre-therapy concerns in cognitive processing speed and working memory, yet only three of six patients who experienced a CNS DLT were detected as having baseline neurobehavioral concerns (Extended Data Table 5). The two adults with grade 4 CNS AEs were not found to have significant baseline neurobehavioral concerns. Neurobehavioral screenings did identify that patients with CNS DLTs experienced a decline or were at risk of decline in neurobehavioral functioning 1–2 courses preceding the actual CNS DLT (see Methods for definition of ‘at risk of decline’ or ‘decline’), with neurobehavioral screens normalizing upon dose hold (Extended Data Table 5).

### Pharmacokinetic assessment

**Fig. 2:**
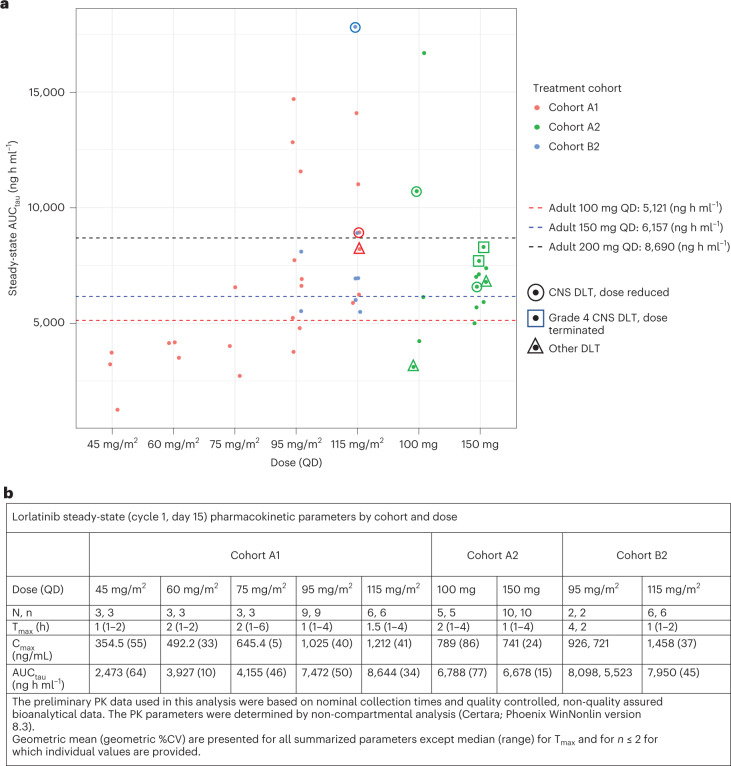
PK analysis of lorlatinib exposure. **a**, Lorlatinib AUC_tau_ at steady state (cycle 1, day 15) compared to adult NSCLC levels. Dots represent individual patient AUC_tau_. Cohort A1 (<18 years), cohort A2 (>18 years) and cohort B2 (<18 years, lorlatinib in combination with topotecan/cyclophosphamide). **b**, Lorlatinib exposure at steady state summarized descriptively by cohort/dose. As shown, no differences in PK were observed for patients on B2 who received lorlatinib with chemotherapy. Although a patient on A1 with the highest C_max_ and steady-state exposure for lorlatinib was a 12-year-old on DL5 who had a CNS DLT, there were six patients on A1 with steady-state exposures 1–2× higher than the adult 200-mg dose with no DLTs observed. CV, coefficient of variation; QD, once a day. Source data

Full plasma pharmacokinetic (PK) parameter values at steady state (cycle 1, day 15) across all dose levels are summarized descriptively in Fig. 2. AUC_tau_ (area under plasma concentration time curve/dosing interval) and C_max_ (maximum plasma concentration) generally increased with increasing dose levels in pediatric patients. Lorlatinib steady-state exposures observed in cohorts A1/B2 at DL4 and DL5, and cohort A2 at 100 mg and 150 mg daily, were in the range of exposures observed in adult patients with lung cancer at 100 mg and 200 mg daily dose levels, respectively. Notably, no differences in AUC_tau_ or C_max_ were observed at steady state for the 17 patients who received the oral dispersion formulation of lorlatinib, and the two adult patients on A2 with CNS DLTs requiring therapy termination had exposures within the range between the NSCLC 100 mg RP2D and 200 mg doses (Fig. 2a).

### Response

**Fig. 3:**
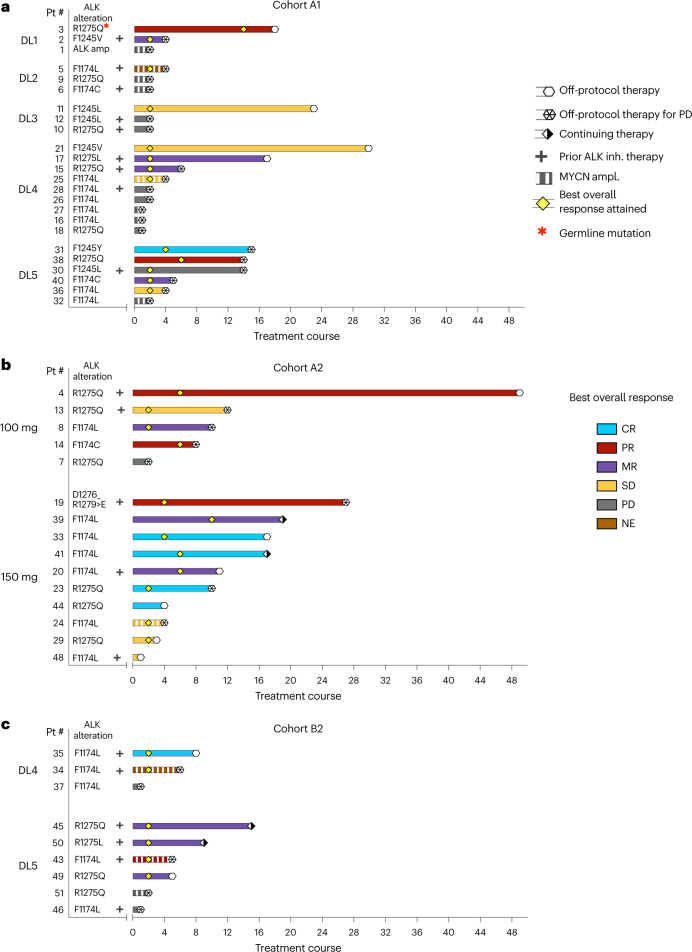
Response characteristics in patients with *ALK*-mutated or *ALK*-amplified neuroblastoma who are receiving lorlatinib as a single agent or in combination with topotecan/cyclophosphamide. The three panels show response onset and duration by NANT criteria^26^ for patients treated with single-agent lorlatinib in cohorts A1 (≥12 months to <18 years), A2 (≥18 years) and B2 (<18 years, in combination with chemotherapy). The color of the bar represents the patient’s BOR; the length of the bar represents duration of response; and the tick mark represents time to best response. Patient 3 (A1, DL1) was the only patient with a germline *ALK* mutation. Patient 42 (enrolled in ongoing B1 lorlatinib monotherapy expansion) and patient 47 (enrolled in ongoing chemotherapy combination for ≥18 years of age) are not included in this evaluation. NE, not evaluable; Pt, patient. Source data

The median number of courses received on cohort A1 was four (range 1–30), with no patients remaining on protocol therapy. The best overall response (BOR) of CR or PR using the NANT criteria^26^ (Methods) for all evaluable patients in cohort A1 was 13% (3/23; 95% confidence interval (CI): 3–34%), including two PR and one CR (Fig. 3a and Extended Data Table 6), with two of these responses, including the CR, occurring at DL5—the cohort A1 RP2D. Four additional patients on cohort A1 had MRs, defined as a CR and/or PR for one parameter (that is, soft tissue tumor, bone or bone marrow), with response of stable disease (SD) for a second parameter and no progressive disease (PD) for the third parameter (NANT response criteria version 2.0 (ref. ^26^)). Based on international consensus that these MRs are biologically and clinically meaningful^29^, an ad hoc analysis showed that the cohort A1 modified RR (BOR of CR/PR/MR by NANT criteria^26^) was 30% (95% CI: 13–53%). For the 11 of 23 evaluable patients who achieved a response of SD or better, median time to BOR was two courses (Fig. 3). Responses were seen across all *ALK* mutations and all ages (2–17 years). Most patients <18 years of age with *MYCN-*amplified/*ALK-*mutated neuroblastoma (6/7, 86%) had PD before or by the end of course 2 (Fig. 3), whereas one patient had MR. Four of nine (44%) patients pre-treated with ALK TKI achieved MR or SD.

The median number of courses received on cohort A2 was ten (range 1–49), with two patients still receiving protocol therapy. The RR (BOR of CR/PR) was 47% (7/15; 95% CI: 21–73%), including four CR and one PR at the RP2D, and modified RR (BOR of CR/PR/MR) was 67% (10/15; 95% CI: 38–88%), with only one patient having PD as best response. For 14 of 15 evaluable patients who achieved SD or better, the median time to BOR was four courses (Fig. 3b and Extended Data Table 6). Responses were seen at all dose levels, across all *ALK* mutations and in three of five (60%) patients with prior ALK TKI. Details about prior ALK TKI therapy can be found in Extended Data Table 7. The one adult with *MYCN-*amplified neuroblastoma treated at 150 mg had best response of SD.

The median number of courses received on cohort B2 was five (range 1–16), with one patient still receiving protocol therapy. The RR (BOR of CR/PR) was 25% (2/8; 95% CI: 3–65%) and modified RR (BOR of CR/PR/MR) of 62% (5/8; 95% CI: 24–91%) for patients who received lorlatinib in combination with topotecan/cyclophosphamide (Fig. 3c and Extended Data Table 6). The median time to BOR of SD or better was two courses. In contrast to the limited response observed for patients with *MYCN-*amplified disease on cohort A1, two of three patients with *MYCN* amplification achieved a PR to lorlatinib plus topotecan/cyclophosphamide.

**Fig. 4:**
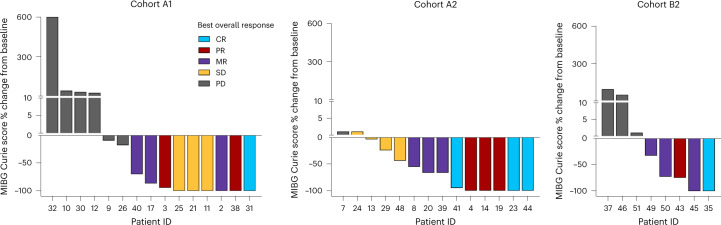
Efficacy of lorlatinib in patients with MIBG avid relapsed or refractory ALK-driven neuroblastoma. Shown are waterfall plots summarizing the best percentage change from baseline in MIBG avidity (change in overall MIBG Curie score^47^ (soft tissue and bone avid lesions) from baseline MIBG Curie score). Bar color represents BOR according to NANT response criteria 2.0 (ref. ^26^). Cohort A1: patients ≥12 months to <18 years of age treated at all DLs with single-agent lorlatinib; cohort A2: patients ≥18 years of age treated with single-agent lorlatinib; cohort B2: patients <18 years of age treated with combination lorlatinib and topotecan/cyclophosphamide. Source data

**Extended Data Fig. 1:**
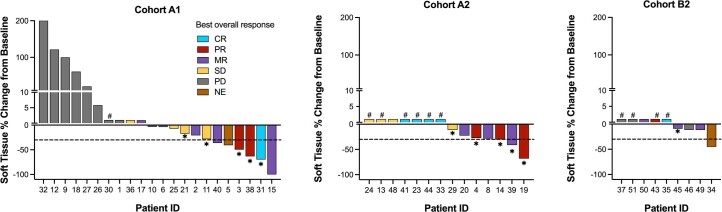
Antitumor activity of lorlatinib by RECIST response in patients with soft tissue disease. Bar color represents best overall response for all sites of disease by NANT response criteria v2.0. Dashed line represents 30% change from baseline to obtain PR or greater in soft tissue sector of response, ^#^Soft tissue not involved at enrollment, *Soft tissue became MIBG non-avid. Source data

**Extended Data Fig. 2:**
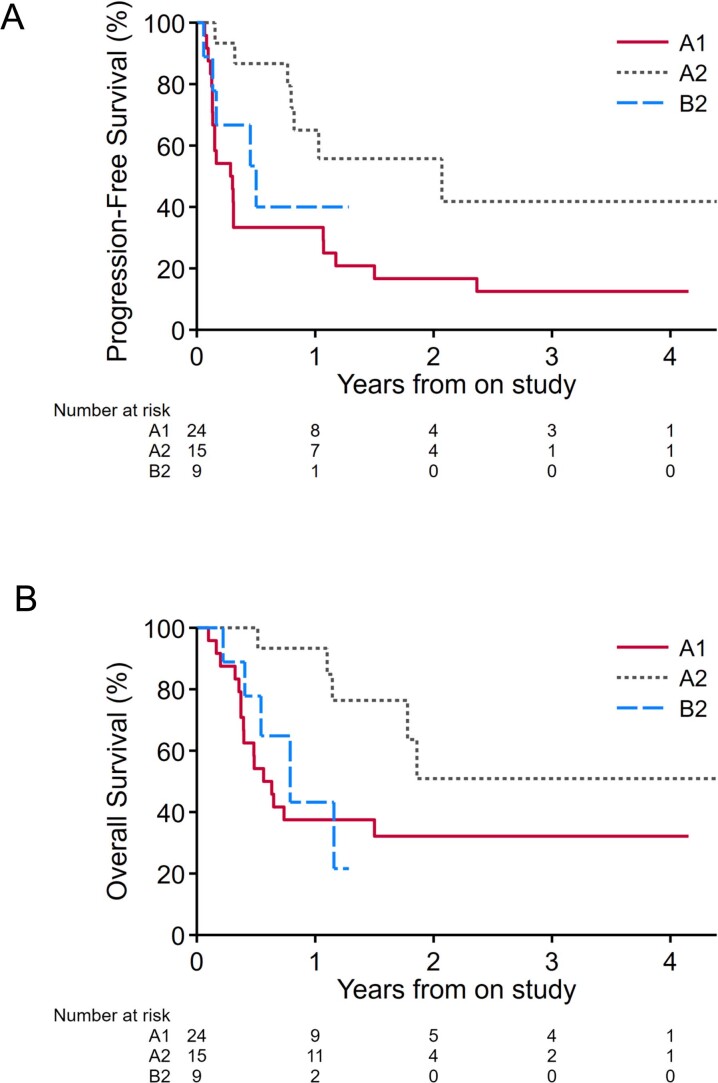
Kaplan-Meier plots of progression-free survival (A) and overall survival (B) by cohort. Source data

NANT response criteria combine anatomical imaging (Response Evaluation Criteria in Solid Tumors (RECIST)) with functional imaging, using ^123^I-metaiodobenzylguanidine (MIBG) scintigraphy or fluorodeoxyglucose-positron emission tomography (FDG-PET) in those patients whose tumors are not MIBG avid, to assess response in soft tissue and bone sites of disease. Across all cohorts, we observed significant improvement in functional imaging (meeting criteria for PR or CR) that exceeded the BOR, primarily because of a lack of sufficient change in soft tissue RECIST measurements. Of the 22 patients with MIBG or PET-avid soft tissue target/non-target lesions and a BOR of SD/MR/PR in part due to incomplete resolution of soft tissue by RECIST (Extended Data Fig. 1), ten (45%) had complete resolution of metabolic activity in soft tissue lesions by MIBG/FDG-PET. Figure 4 further demonstrates the functional imaging responses observed in each cohort, where patients with BOR of SD/MR/PR demonstrated a complete resolution of MIBG avidity (CR) in bone and/or soft tissue in five of eight (63%) on A1, three of ten (30%) on A2 and one of four (25%) on B2 (Supplementary Table 2). The median duration of follow-up for progression-free survival and overall survival for the three cohorts are shown in Extended Data Fig. 2, highlighting the differences between cohorts A1 and A2.

**Extended Data Fig. 3:**
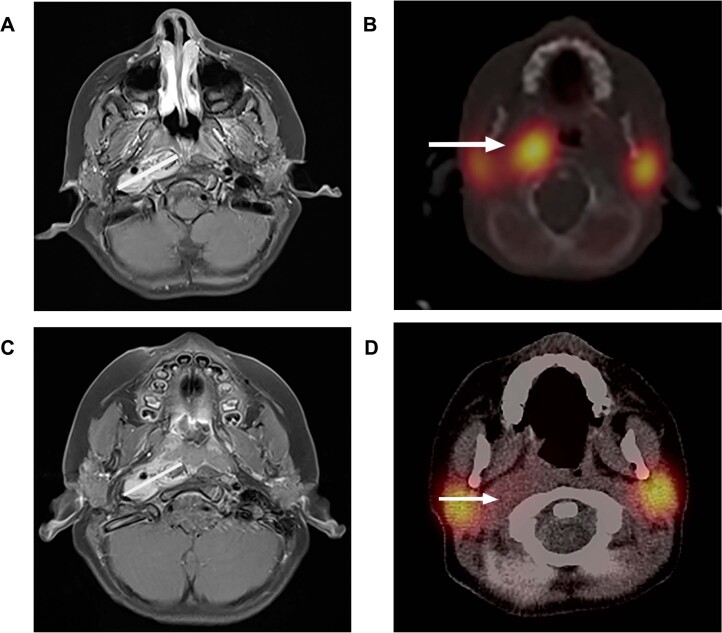
Images of a representative patient. **Enrollment Images: a**) Axial MRI T1 fat-saturated image post contrast shows an enhancing right retropharyngeal mass with white line indicating maximum transverse diameter. **b**) Axial ^123^I-MIBG SPECT CT fused image shows marked MIGB uptake in the mass (white arrow). **Imaging six months after the initiation of therapy: c**) Axial MRI T1 fat-saturated image post contrast shows an enhancing right retropharyngeal mass with white line indicating no significant change in maximum transverse diameter consistent with stable disease by RECIST 1.1 criteria. **d**) Axial ^123^I-MIBG SPECT CT fused image shows resolution of MIBG uptake in the mass (white arrow). Normal salivary gland uptake of ^123^I-MIBG bilaterally is seen.

Two patient vignettes highlight the heterogeneous and persuasive metabolic responses to lorlatinib monotherapy. Patient 3 treated at DL1 on cohort A1 had a germline *ALK* R1275Q mutation with metastatic primary refractory neuroblastoma. While receiving lorlatinib monotherapy, the patient had BOR of PR with a CR in bone marrow by course 2, near CR by MIBG Curie score (Fig. 4) and PR in soft tissue (Extended Data Fig. 1). This patient went on to receive 18 courses of therapy, after which the patient came off protocol therapy for significant weight gain. Patient 11 in cohort A1 with a somatic *ALK* F1245V mutation and primary refractory disease treated at DL3 had a large MIBG avid soft tissue mass surrounding the right cervical internal artery (Extended Data Fig. 3a,b). After four cycles of lorlatinib, the tumor became MIBG non-avid (Extended Data Fig. 3d) despite stable measurements by RECIST (Extended Data Fig. 3c and Extended Data Fig. 1), consistent with BOR of SD. The patient received 20 courses of lorlatinib and was removed from protocol therapy to receive local control with external beam radiation and has remained on commercial lorlatinib for 4.5 years with MIBG-negative disease.

## Discussion

Despite an exponential increase in discoveries related to neuroblastoma genomics and immune evasion, a child diagnosed with high-risk neuroblastoma today is subjected to a largely empiric and vastly intensive regimen of genotoxic chemotherapeutics, radiation and GD2-targeted immunotherapy that, although effective, has substantial acute and long-term side effects^30^. We have sought to change this paradigm by developing and implementing biomarker-directed precision therapies. In this first-in-child phase 1 study of lorlatinib given alone or in combination with chemotherapy, lorlatinib was found to be safe and tolerable in pediatric, adolescent and adult patients with relapsed or refractory *ALK-*mutated or *ALK*-amplified neuroblastoma.

The RP2D of 115 mg/m^2^ daily in patients <18 years of age was about twice the regulatory-approved adult NSCLC starting dose, as predicted by pre-clinical studies of neuroblastoma harboring *ALK* mutations^25^. The clinical studies of crizotinib and ceritinib for patients with refractory or relapsed ALK-driven neuroblastoma showed responses that were limited mainly to tumors harboring an ALK R1275 mutation with no activity in tumors harboring an F1174 mutation^12,31^. Single-agent lorlatinib demonstrated clinical activity across patients of all ages harboring the three neuroblastoma-specific hotspot *ALK* mutations, including patients who had previously received other ALK TKIs. Remarkably, the only patient with a germline *ALK* mutation who was treated at DL1 with widely metastatic chemo-refractory disease had an outstanding and sustained response to lorlatinib, similar to the responses seen with crizotinib and ceritinib in the same context^12,31^, providing further proof of concept that ALK inhibition in the context of a germline mutation is highly effective. Notably, adolescents and adults with ALK-driven neuroblastoma had objective and sustained responses, an important outcome for patients who often have an indolent clinical course with de novo chemotherapy resistance and an abysmal overall outcome^32–35^. Genomic correlates of indolent neuroblastoma, such as somatic alterations in the ATRX gene or activation of the alternative lengthening of telomeres (ALT) mechanism, have been defined^36–41^, but we have been unsuccessful at leveraging these discoveries into more precise therapies. Future studies looking at ALK status in conjunction with an ALT phenotype will be integral for bringing new therapies to these patients.

Overall, younger patients treated with lorlatinib monotherapy, in particular those also harboring *MYCN* amplification, had fewer responses than older patients despite similar PK exposure. There is a significant correlation in the A1 cohort between age at study entry and response, but this is clearly confounded by *MYCN* amplification status (Supplementary Fig. 1). The ongoing Childrenʼs Oncology Group ANBL1531 phase 3 trial and planned European trial also integrating lorlatinib into frontline therapy will provide more definitive data on any relationship between age and anti-tumor activity and will interrogate ALT status for all enrolled patients. *MYCN* amplification is a truncal event in neuroblastoma that is almost never detected in tumors of patients >12 years of age, and it portends an especially aggressive disease course at relapse^1^. We posit that, due to intra-tumor heterogeneity and clonal evolution, mutations in *ALK* are branched events in these patients and that lorlatinib monotherapy will be insufficient^42,43^. Notably, we observed objective responses in two patients with *MYCN* amplification treated with lorlatinib in combination with chemotherapy, suggesting that *MYCN*-amplified, ALK*-*mutant disease can potentially be overcome with the addition of chemotherapy. We posit that future development of a strategy of lorlatinib in combination with, for example, a MYCN-directed protein degrader may be especially impactful for this patient population.

In the context of this phase 1 trial of safety and tolerability, we observed objective responses, and, of those, patients who achieved objective responses of CR/PR to lorlatinib as well as those with MR/SD went on to receive multiple courses of therapy owing to sustained responses. These response rates to lorlatinib compare favorably to previous trials of first-generation and second-generation ALK inhibitors crizotinib (15% CR/PR) or ceritinib (20% CR/PR) in children with ALK-driven neuroblastoma^12,13^. Notably, responses to lorlatinib monotherapy were seen across all hotspot mutations, as opposed to crizotinib and ceritinib where primary resistance was observed in common neuroblastoma mutations F1174 and R1245. Certain key characteristics, such as MYCN status and prior ALK TKI exposure, were not specified in those trials, making it difficult to further compare efficacy.

Although historical trials report CR/PR rates, there is now international consensus that minor response be used given the importance of MIBG response. Some patients with BOR of MR/SD were notable for having CR by functional imaging (MIBG or PET becoming non-avid) and bone marrow CRs achieved, with halted soft tissue tumor growth control that was not reflected in the BOR. In the era of targeted therapies such as lorlatinib, we are recognizing that durable metabolic responses result in sustained tumor control, supporting why the international community has added minor response in the overall response assessment. We speculate that *ALK* mutations may contribute to a differentiation block and that potent ALK TKI therapy can cause tumors to terminally differentiate, which is why response determination using RECIST may fall short in demonstrating the full effects of ALK TKIs. These data support that novel response criteria may be necessary to understand the anti-tumor activity of molecularly targeted agents.

The safety profile of lorlatinib across all ages was similar in scope and grade to those reported in NSCLC studies, with the unique toxicities of lorlatinib, such as weight gain and increased circulating lipids, manageable with supportive care, diet management and statins. Only one patient, who had a germline *ALK* mutation, discontinued lorlatinib owing to significant weight gain. AEs such as peripheral neuropathy, peripheral edema and CNS effects reported in adults with ALK fusion NSCLC^20–22^ were more frequently observed in adults with neuroblastoma compared to children. Although superior responses were observed at the 150-mg dose (four CRs), the A2 cohort is ongoing at 100 mg to determine whether the established RP2D in adults with neuroblastoma can be further refined. Patients <18 years of age treated with lorlatinib monotherapy overall had less toxicity, with only 9% requiring a dose reduction. Lorlatinib in combination with topotecan/cyclophosphamide was well tolerated and showed no overlapping toxicities in patients <18 years of age, allowing for the monotherapy RP2D to be combined with standard-of-care chemotherapy.

Lorlatinib was optimized to penetrate the blood–brain barrier, leading to improved CNS distribution as well as intracranial responses in patients with NCSLC with brain metastases^19,20^. Although this contributes to the CNS effects seen in adults with neuroblastoma enrolled on this study, neuroblastoma has a propensity for recurrence in the brain, especially after anti-GD2 antibody therapy that does not cross the blood–brain barrier^44^; thus, lorlatinib’s CNS penetration is an advantageous attribute. Intracranial anti-tumor activity could not be assessed in our study as no patient with CNS metastases enrolled; however, proof of concept has been demonstrated in a child with ALK-fusion-positive glioma^45^. Real-time neuropsychological surveillance on our study identified the presence of baseline (that is, pre-therapy) neurobehavioral symptoms across patients of all ages, including those who did and did not develop CNS effects, suggesting challenges in differentiating and predicting later CNS effects via pre-treatment neurobehavioral screening methods in this patient population. Serial neuropsychological monitoring detected increased risk for neurobehavioral decline in all patients who experienced a CNS DLT and assisted with their Common Terminology Criteria for Adverse Events (CTCAE) grading. However, risks for neurobehavioral decline were also identified in patients who did not have a CNS AE. Therefore, future studies examining the sensitivity and specificity of neurobehavioral screening measures to detect change in neurocognitive functioning in response to lorlatinib in pediatric patients are warranted, considering the limitations and feasibility of real-time monitoring within the context of a larger clinical trial.

Grade 4 CNS AEs were limited to two adult patients, with one having a prior undisclosed psychiatric history, supporting the continued need for strict eligibility criteria to exclude those patients who are most at risk. Failure of certain patients to disclose prior psychiatric clinical history has also highlighted the need to identify more robust objective mechanisms to screen for pre-treatment psychiatric risk. Most importantly, CNS effects consistently improved with dose hold and reduction, allowing most to continue lorlatinib to benefit from its potent anti-tumor effects. Lorlatinib exposure was not affected by age nor by addition of chemotherapy.

The source of tumor tissue used for *ALK* gene sequencing was variable, with some from tumor obtained before relapse and others from tissue biopsies performed at relapse yet not at time of enrollment. These single tumor biopsies are inadequate representations of the entire disseminated tumor bulk, and, therefore, sequencing data used for enrollment may not represent the intra-tumor heterogeneity and clonal evolution at study entry. We, therefore, collected research-based serial ctDNA to characterize genomic changes in response to therapy, detect early disease progression and identify evolving alterations driving tumor heterogeneity and lorlatinib resistance. This same assay has already informed on the genetic evolution of relapsed neuroblastoma for patients not on this trial^46^. The full landscape of ctDNA genomic alterations in patients with ALK-driven refractory or relapsed neuroblastoma treated on this trial is detailed in a companion manuscript.

The current therapeutic paradigm for patients with high-risk neuroblastoma is to treat with an intense and largely empiric multi-modal approach. The discovery of ALK as a biomarker of inferior prognosis and of these oncogenic mutations as drivers of tumorigenesis in a significant subset of newly diagnosed patients has shifted the paradigm to study ALK inhibition in frontline therapy. Based on the results of this phase 1 study, which supports the hypothesis that lorlatinib will be safe and effective for patients with newly diagnosed ALK-driven, high-risk neuroblastoma, we recently implemented a major amendment to replace crizotinib with lorlatinib in the Childrenʼs Oncology Group phase 3 trial (NCT03126916). Incorporation of lorlatinib in the European phase 3 trial in collaboration with the International Society of Paediatric Oncology European Neuroblastoma (SIOPEN) is planned as well. Integration of a potent targeted therapy in the treatment of newly diagnosed patients with *ALK-*aberrant, high-risk neuroblastoma holds substantial promise for improving patient outcomes.

## Methods

### Trial oversight

This trial was designed by the trial study committee and the trial sponsor investigator (Araz Marachelian, NANT Medical Director)—registration number NCT03107988. The trial was designed in collaboration with Pfizer, which provided lorlatinib and funding for the trial. Trial investigators provided input into the trial. Each site’s institutional review board approved the protocol and consent (Children’s Hospital Los Angeles Institutional Review Board; Emory University Institutional Review Board; Children’s Hospital of Philadelphia Institutional Review Board; Cincinnati Children’s Hospital Institutional Review Board; Colorado Multiple Institutional Review Board; Cook Children’s Health Care System Institutional Review Board; Comité de Protection des Personnes Ile-de-France X; Dana-Farber Cancer Institute, Office for Human Research Studies; London City & East Research Ethics Committee; Seattle Children’s Institutional Review Board; SickKids Research Ethics Board; University of Chicago Biological Sciences Division Institutional Review Board; University of California, San Francisco Human Research Protection Program Institutional Review Board; and University of Michigan Medical School Institutional Review Board). The trial was conducted in accordance with the Declaration of Helsinki, International Conference on Harmonization guidelines for Good Clinical Practice and local regulations. Patients or legal guardians provided informed consent, and assent was obtained per institutional guidelines. Participants were not compensated. Each local IRB/IEC (where required by the IRB/IEC) were notified of all serious, unexpected adverse drug reactions involving risk to human patients. Progress reports were also provided to IRBs/IECs. The NANT Consortium independent Data and Safety Monitoring Board monitored the study. The sponsor had full access to the study as it was an open-label study. The sponsor and authors jointly collected and analyzed the data. All authors participated in the writing of the report and had full access to the raw data. The first and corresponding authors had full access to all the data in the study and had final responsibility for the decision to submit for publication. All authors vouch for the validity of the trial results and adherence to the protocol.

### Participants

Patients ≥12 months of age with no upper age limit containing a tumor somatic *ALK* alteration were eligible if they met one of the following responses to prior frontline high-risk therapy: relapsed disease; refractory disease (persistent disease after BOR of SD after minimum of four induction cycles); or persistent disease (persistent disease after BOR of PR after minimum of four induction cycles). Patients had to meet criteria for presence of neuroblastoma tumor 4 weeks before enrollment in at least one of the following sites: soft tissue (at least one target or non-target lesion); bone (minimum of one site of disease by functional imaging using MIBG or FDG-PET); or presence of tumor in bone marrow (tumor detected in bone marrow biopsies or aspirates). Eligible *ALK* somatic aberrations included an *ALK-*activating mutation, *ALK* amplification (>10 signals of the *ALK* gene) or the presence of any *ALK* fusion that arises from a chromosomal translocation. Patients had to be fully recovered from prior therapy, including a minimum of 2 weeks off chemotherapy; 7 days off biologic therapy; the longer of 7 days or three half-lives off monoclonal antibody; 2 weeks off radiation, except large field that required 12-week washout; and 6 weeks from prior autologous stem cell transplant. Prior ALK inhibitor therapy, except for prior lorlatinib, was allowed, and active CNS disease was allowed. All patients met standard organ function criteria before enrolling. Patients were excluded if they were pregnant, breastfeeding, on hemodialysis, had an active or uncontrolled infection or had a known chronic severe psychiatric disorder or any history of suicidal ideation or attempt.

### Trial design: clinical considerations

We conducted a phase 1 international multi-center study in the NANT Consortium of lorlatinib both as single agent and in combination with chemotherapy in patients with *ALK-*aberrant relapsed, refractory or progressive neuroblastoma. Lorlatinib was administered orally once daily for 28 days per course in both the single-agent and chemotherapy combination cohorts. Patients ≥12 months of age and <18 years of age and <1.73 m^2^ body surface area (BSA) for DL1 and DL2, <1.43 m^2^ BSA for DL3 and <1.56 m^2^ BSA for DL4 were assigned to the pediatric and adolescent cohort A1, where lorlatinib was given as a single agent. Five DLs (45, 60, 75, 95 and 115 mg/m^2^/dose) were assessed in A1, using a 3 + 3 design. Patients ≥18 years of age or ≥ corresponding BSA noted above for DL1, DL2, DL3 and DL4 were assigned to cohort A2, where two doses of single-agent lorlatinib at 100 mg (the RP2D for adult NSCLC^21^) and 150 mg daily were assessed. Patients ≥12 months to <18 years of age who received lorlatinib in combination with chemotherapy were assigned to cohort B2. B2 patients received fixed doses of topotecan (0.75 mg/m^2^/dose intravenously (IV)) and cyclophosphamide (250 mg/m^2^/dose IV) on days 1–5 of each course (topotecan/cyclophosphamide) in combination with two escalating doses of lorlatinib (95 mg/m^2^/dose or 115 mg/m^2^/dose daily on days 1–28 of each course). Myeloid growth factor was given 24 hours after the completion of topotecan/cyclophosphamide on B2, according to institutional standards. Instructions and hands-on training for a tablet dispersion formulation, validated by Pfizer, was provided to families of patients who could not swallow tablets. In the absence of disease progression or meeting off-protocol criteria, patients were allowed to continue lorlatinib indefinitely. Cohort B2 opened at DL4 once cohort A1 DL4 was deemed tolerable and safe. B2 did not dose escalate to DL5 (115 mg/m^2^) until both cohort B2 DL4 in chemotherapy combination and cohort A1 DL5 (115 mg/m^2^) as single agent was deemed tolerable and safe.

### Trial design: objectives

The primary objectives were to determine the safety, pharmacokinetics and RP2D of lorlatinib both as a single agent and in combination with topotecan/cyclophosphamide when administered orally to children, adolescents and adults with relapsed or refractory high-risk neuroblastoma with tumor containing a confirmed pathogenic *ALK* fusion protein, *ALK* mutation or *ALK* amplification. Additional objectives were to preliminarily evaluate the anti-tumor activity of lorlatinib with or without chemotherapy and serially evaluate patient ctDNA to determine the profile of acquired somatic mutations.

### Trial design: statistical methods

In this phase 1 trial, evaluation of lorlatinib DLs followed the 3 + 3 dose-escalation design in each of the three cohorts separately. Hence, at each DL during the escalation portion, the plan was to enroll either three or six patients who were evaluable for DLT, with six DLT-evaluable patients at the maximum tolerated dose (MTD) or highest DL planned. In this trial, two exceptions occurred. In cohort A1 at DL4, because two of the first five patients were inevaluable for DLT assessment (due to disease progression before starting the second course, although neither had experienced DLT in the first course), the Study Management Committee (SMC) decided to expand DL4 to two additional patients to further evaluate later course toxicities before escalating to DL5. Once none of the first five DLT-evaluable patients experienced DLT during the DLT observation period, the SMC decided to open DL5 (Table 2 and Fig. 1c). The second exception occurred in cohort A2 at the first DL of 100 mg. Initially, this was the only DL planned, but, with a subsequent amendment, a second DL was added to cohort A2 (150 mg). At that time, because all five patients enrolled at 100 mg were DLT evaluable and none had experienced DLT during the DLT observation period, the SMC decided to assign the next A2 patient to the 150-mg DL. Finally, because of the nature of the patients in cohort A2 (which had only two planned DLs), an expansion of up to six additional patients was planned for the highest DL tested with zero or one DLT in six DLT-evaluable patients. Accrual could continue in the expansion cohort as long as none, or at most one, of the patients experienced DLT in the DLT observation period; if two or more patients experienced DLT in the DLT observation period, this would lead to a discussion of the suitability of this dose. The probability of observing more than two patients with DLT, out of six patients, is 0.11, 0.22, 0.47, 0.68 and 0.77 when the true probability of DLT is 0.10, 0.15, 0.25, 0.35 and 0.40, respectfully.

All DLTs in the first course of therapy and CNS DLTs through the second course of therapy had an impact on decisions regarding dose escalation due to delayed CNS toxicities observed in the adult lorlatinib trials^20,21^. In cohorts A1 and A2, patients were deemed evaluable for toxicity and contributed to dose-escalation purposes if they received a minimum of 75% of the planned lorlatinib doses in courses 1 and 2 or if they experienced any DLT during course 1 or a CNS DLT in courses 1 or 2. For patients in cohort B2, patients were evaluable for dose-escalation purposes if they had received at least 75% of the planned lorlatinib, cyclophosphamide and topotecan doses in course 1 or if they had a course 1 DLT. Patients who were not evaluable for DLT were replaced for dose-escalation purposes. All eligible patients who received any amount of lorlatinib were considered evaluable for response unless deemed by the SMC to have inadequate imaging to assess overall response. The MTD was defined as the highest DL tested at which zero of six or one of six patients experience DLT. After determination of the MTD and review of all toxicities, and provided that other safety considerations were acceptable, the MTD was labeled the RP2D.

Standard statistical methods were used to summarize data; exact Clopper–Pearson CIs are reported for binomial probability or corresponding percentages. Analyses were performed with SAS software version 9.4. The cutoff for toxicity and response data for patients reported in this manuscript was 13 September 2022.

### Toxicity assessment

Toxicity was graded according to CTCAE version 4. DLT definitions included only toxicities deemed at least possibly related to therapy. For A1 and A2, hematologic DLT was defined as grade 4 thrombocytopenia or grade 4 neutropenia >7-day duration or a delay in the start of subsequent course by more than 14 days due to ongoing thrombocytopenia or neutropenia, in the absence of bone marrow disease progression seen on clinically indicated bone marrow biopsy (if performed). For cohort B2, hematological DLT is defined as a delay in the start of subsequent course by more than 14 days due to ongoing thrombocytopenia or neutropenia, in the absence of bone marrow disease progression seen on clinically indicated bone marrow biopsy after the first dose reduction of cyclophosphamide and topotecan. Non-hematologic DLT was defined as any non-hematologic toxicity that delayed the start of a subsequent cycle by more than 14 days or any grade ≥3 toxicity, with the exception of the following grade 3 toxicities: nausea, vomiting, anorexia or dehydration resolving to grade ≤2 within 72 hours; increase in hepatic transaminase or electrolyte abnormality resolving eligibility levels within 7 days; diarrhea persisting for less than 72 hours; fever; infection; febrile neutropenia; high cholesterol or triglycerides if responsive (to ≤grade 2) with statin treatment; weight gain; and hyperglycemia if responsive (to <grade 2) with dietary modification. Additional non-hematologic DLT included any grade 2 non-hematological toxicity that persists for more than 7 days and is considered sufficiently medically significant or sufficiently intolerable by patients that it requires treatment interruption; recurrence of grade 2 neuropsychological effects; grade 2 radiologically confirmed pancreatitis; recurrence of grade 2 first-degree symptomatic heart block; and grade 1 second-degree (Mobitz type 1 or 2) asymptomatic heart block.

### CNS effects monitoring

Patients on this first-in-child study of lorlatinib underwent routine real-time neurobehavioral monitoring at baseline and all disease evaluation timepoints as part of the required observations using various neurodevelopmental assessments, computerized cognitive measures and behavioral rating questionnaires dependent upon age (see Supplementary Table 3 for neuropsychological procedures by age group):

The *Adaptive Behavior Assessment System* 3rd edition (ABAS-3; Harrison & Oakland, 2015) parent-report and self-report forms were used for the assessment of adaptive skills for individuals across all ages. Internal consistency has been reported to be 0.96 or greater for the composite adaptive behavior scale and between 0.86 and 0.99 for the subscales. Construct, convergent and discriminant validity have also been established for this measure.

The *Behavior Assessment System for Children* 3rd edition (BASC-3; Reynolds & Kamphaus, 2015) is a questionnaire assessing behavioral, emotional and adaptive functioning across settings. For purposes of this study, the Parent Rating Scales (PRS) were administered for patients ages 2–5 years (Preschool form, PRS-P), 6–11 years (Child form, PRS-C) and 12–17 years (Adolescent form, PRS-A). The Self-Report of Personality (SRP) College Form was administered for adult patients ages 18–25 years. The reliability of the BASC-3 is strong, with internal consistency averaging above 0.80 for all the age-specific versions of this questionnaire, and average test–retest reliability is 0.86.

The *Behavior Rating Inventory of Executive Function* (BRIEF; Gioia et al. 2000) is a questionnaire designed to assess behavioral manifestations of executive functioning and includes multiple versions. Parents of participants aged 2–5 years completed the 63-item preschool version (BRIEF-P). Parents of participants aged 6–17 years completed the BRIEF parent form, which consists of 86 individual items from which eight clinical scales, two indices and one composite score are derived. Patients who were ≥18 years of age completed a 75-item self-report adult version (BRIEF-A). Scores are standardized by age and gender, and high internal consistency (0.73–0.98) and test–retest reliability (0.76–0.90) were observed. Construct, content, convergent and discriminant validity have been established.

*CogState Research Battery* (version 7; Sands et al. 2017; Heitzer et al. 2018) is a flexible battery of computer-based tests developed to monitor domains of cognition typically affected by cancer treatment, such as attention, memory, executive function and processing speed. Three different versions were used, one for children 3–5 years of age, one for children 6–9 years of age and one for patients 10 years of age and older. Age-standardized *z*-scores were derived based on the CogState normative dataset finalized in September 2014 for adults and in February 2016 for pediatrics, with construct validity, criterion validity, cultural equivalence and limited practice effects established.

Only measures with established language translations were used. If measures were not available in a patient’s primary language, neurobehavioral toxicity assessment was based on the treating physician’s clinical determination using CTCAE version 4.0 grading criteria. All measures were individually administered by trained, qualified site personnel. Standardized scores were obtained for each instrument administered by comparing raw scores to normative samples provided by the test publishers. All evaluation results were reviewed, analyzed and interpreted centrally in real time by a pediatric neuropsychologist. Determination of clinically meaningful change in functioning (that is, significant decline) relative to baseline or previous screening exam was made at each retest interval. This was calculated using reliable change indices (RCIs) for individual tests or scales, which account for standard errors of measurement, to determine whether a statistically significant change in scores occurred between two timepoints. Meaningful change in performance was considered to occur when clinically significant decline (using 90% CI) was present in 50% of select administered measures. Risk for possible decline and consideration for modification of neuropsychological retest interval (increased screening frequency) was determined using a 25% impairment classification model in an effort to reduce potential for type 1 error. A neuropsychological status report detailing findings was generated and provided to the treating oncologist within 72 hours to assist with CNS toxicity grading. A neuropsychology grading tool was used as a tool for physicians to grade patient baseline and subsequent toxicities. The physician assessment used both the neuropsychological report and history to grade neuropsychological toxicities.

### Response evaluation

Patients underwent disease staging at baseline, after courses 2 and 4 and then every four courses thereafter. The primary endpoint for response analysis is the BOR, defined as the best response observed before progression or start of another therapy. All eligible patients who receive any amount of lorlatinib were considered evaluable for response unless deemed by the SMC to have inadequate imaging to assess overall response. Response was graded according to the NANT response criteria version 2.0 (ref. ^26^), modified from the International Neuroblastoma Response Criteria (INRC)^48^. NANT response criteria use three components for assessment of overall response: soft tissue response assessed by anatomical imaging and avidity on MIBG or FDG-PET; bone response assessed by uptake in bone by MIBG or bone involvement on FDG-PET; and bone marrow response assessed by morphology on bilateral aspirates and biopsies. Each response component (soft tissue, bone and bone marrow) are graded as CR, PR, SD and PD. Overall response grading is defined as: CR, if a response of CR in all components that are involved at baseline; PR, if a response of PR or better in all involved components; MR, if a response of PR or better but with at least one involved component as SD and no PD in all involved components; SD, if a response of SD in all involved components; and PD, if a response of PD in at least one involved component. The overall response definitions are identical to overall responses (CR, PR, SD and PD) as defined by the INRC. Response assessment is initially performed by sites and reviewed against source documents by NANT operations. Additional blinded central review of all radiological or pathological data was conducted for patients with overall PR or better and, in addition, radiologic pathological data of all involved sites for patients with MR to assign the final BOR on this study. Response by MIBG was determined using the Curie criteria^49^. RR was determined as the proportion of patients with BOR of CR and PR in each cohort. Based on an international consensus by the National Cancer Institute Clinical Trials Planning Committee to refine tumor site eligibility criteria and evaluation of disease response for early-phase clinical trials in children with high-risk recurrent, refractory and progressive neuroblastoma^29^, we also performed an ad hoc analysis to determine a modified RR that includes the proportion of patients with BOR of CR, PR and MR.

### Pharmacokinetics

Lorlatinib was isolated from human plasma by a liquid–iquid extraction procedure. After evaporation of the organic extract under nitrogen, the residue was reconstituted, and the final extract was analyzed for lorlatinib concentration by liquid chromatography with tandem mass spectrometry (LC–MS/MS) using a previously reported assay (Chen et al. *Adv. Ther.* **37**, 745–758 (2020)). The lower limit of quantitation (LLOQ) for measurement of lorlatinib concentration in human plasma was 2.50 ng ml^−1^, with linearity demonstrated up to 2,500 ng ml^−1^ (upper limit of quantitation (ULOQ)).

PK parameters for lorlatinib were derived by non-compartmental analysis (NCA) using Phoenix WinNonlin version 8.3. A linear-log trapezoidal method was used to calculate AUC. A best-fit method was used to estimate terminal elimination and to extrapolate any plasma concentration versus time profiles to compute AUC_tau_. The PK results were summarized by cohort and DL and graphically presented using R version 3.6.1.

### Reporting summary

Further information on research design is available in the Nature Portfolio Reporting Summary linked to this article.

## Online content

Any methods, additional references, Nature Portfolio reporting summaries, source data, extended data, supplementary information, acknowledgements, peer review information; details of author contributions and competing interests; and statements of data and code availability are available at 10.1038/s41591-023-02297-5.

### Supplementary information

Supplementary Information Supplementary Tables 1–3 and Supplementary Fig. 1

Reporting Summary

### Source data

Source Data Fig. 2 Source data for PK figure and table

Source Data Fig. 2 Source data for PK figure and table

Source Data Fig. 3 Statistical source data for swimmer plot

Source Data Fig. 4 Statistical source data for MIBG Curie score change waterfall plots by cohort

Source Data Extended Data Fig. 1 Statistical source data for soft tissue/RECIST waterfall plots by cohort

Source Data Extended Data Fig. 2 Statistical source data for progression-free survival/overall survival curves

Source Data Extended Data Tables 2, 3 and 4: Statistical source data for AEs by cohort

Source Data Extended Data Table 6 Statistical source data for BOR by cohort

Source Data Extended Data Table 7

## Figures and Tables

**Extended Data Table 1:**
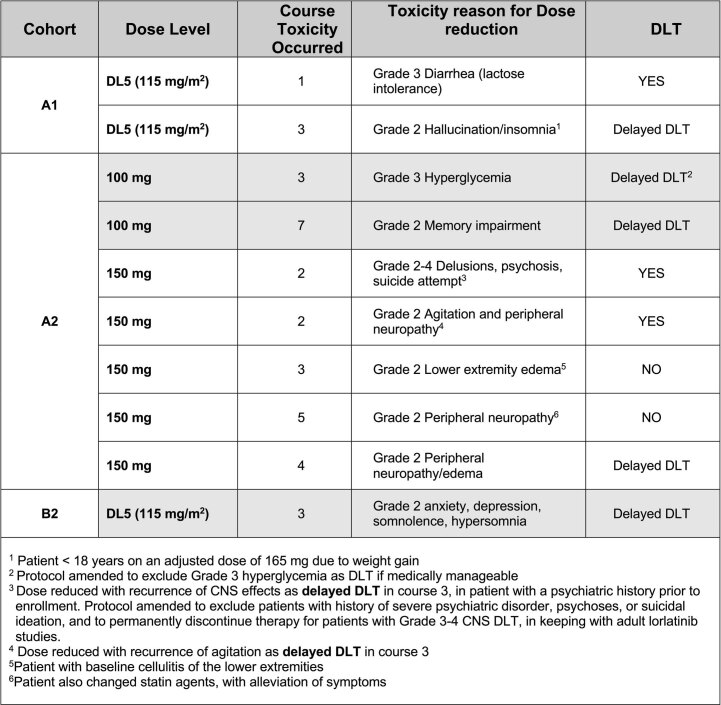
Dose reduction by cohort/DL

**Extended Data Table 2:**
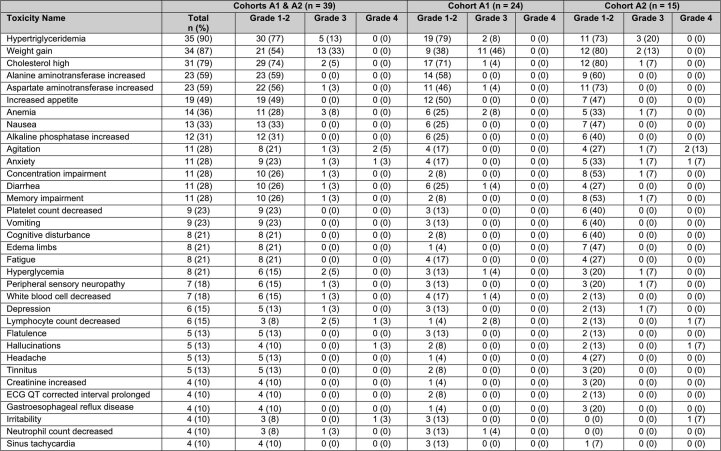
Single-agent lorlatinib treatment-related AEs (A1 and A2)

Treatment-related AEs in at least 10% of patients. Each patient reported once as maximum grade in any course, with attribution possibly, probably and definitely.

Source data

**Extended Data Table 3:**
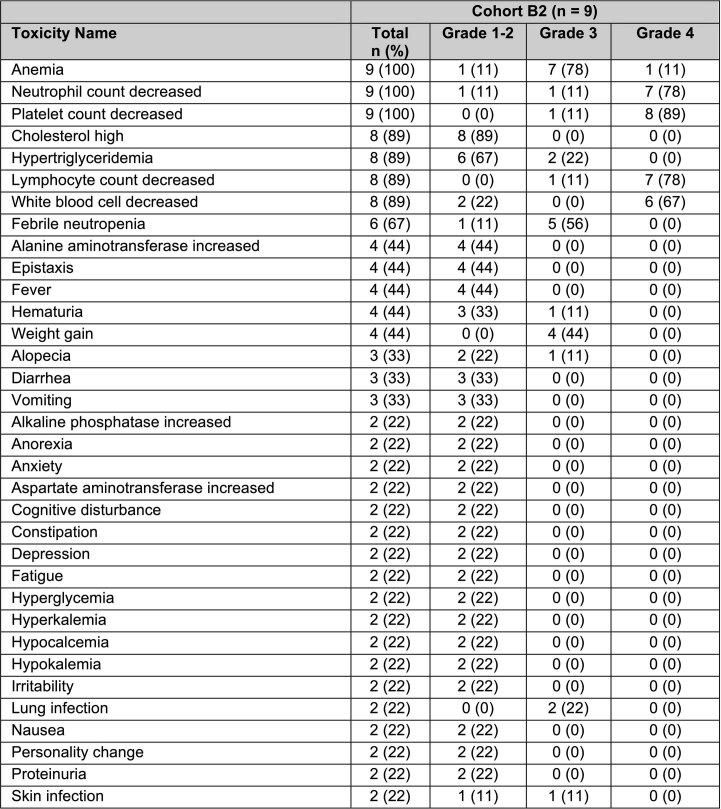
Lorlatinib + chemotherapy in <18 years (cohort B2)

Treatment-related AEs in at least 10% of patients. Each patient reported once as maximum grade in any course, with attribution possibly, probably and definitely.

Source data

**Extended Data Table 4:**
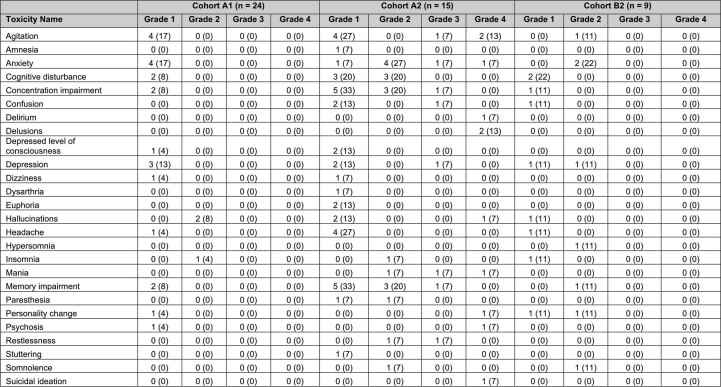
CNS effect treatment-related AEs in all cohorts (attribution possibly, probably and definitely)

Source data

**Extended Data Table. 5:**
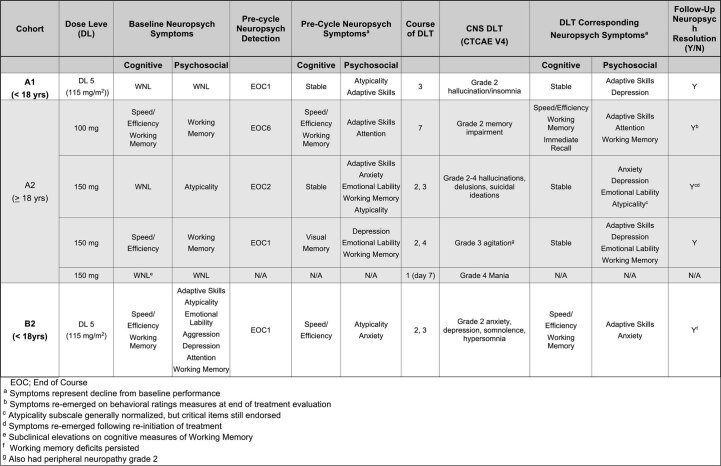
Real-time neuropsychological screening results in patients with CNS DLTs

**Extended Data Table 6:**
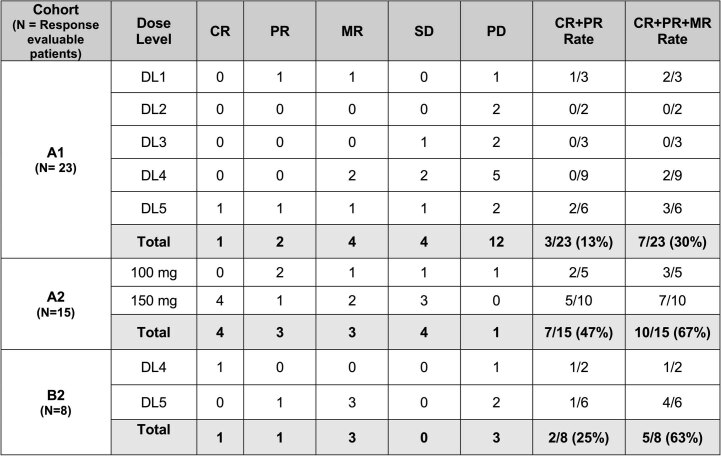
Responses by dose and cohort by NANT criteria^26^

Source data

**Extended Data Table 7:**
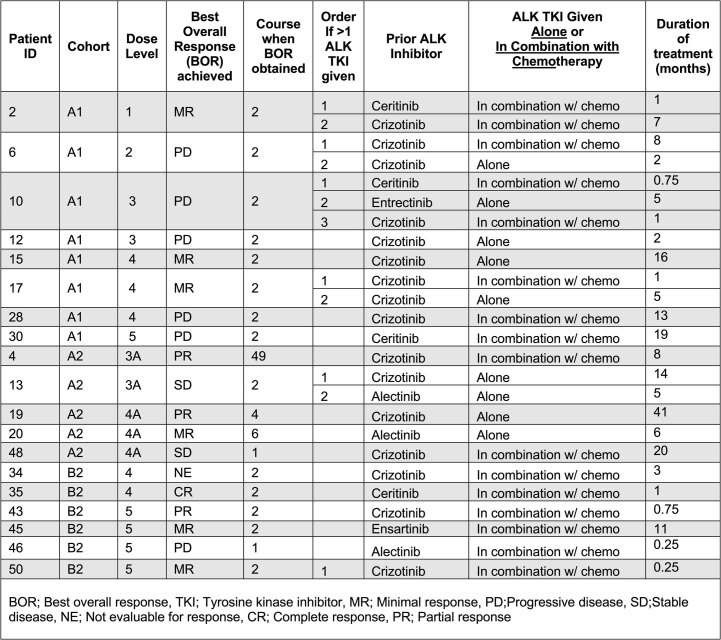
ALK Inhibitor Therapy Prior to Enrollment

Source data

## Data Availability

The anonymized derived data from this study that underlie the results reported in this article will be made available, beginning 12 months and ending 5 years after this article publication, to investigators who sign a data access agreement and provide a methodologically sound proposal to medinfo@blueprintmedicines.com. Source data are provided with this paper.
