# Supplementary material for: Lorlatinib with or without chemotherapy in ALK-driven refractory/relapsed neuroblastoma: phase 1 trial results
Source: Nat Med. 2023 Apr 3;29(5):1092–102. doi: 10.1038/s41591-023-02297-5 (PMC10202811; doi:10.1038/s41591-023-02297-5)

nature medicine

Article https://doi.org/10.1038/s41591-023-02297-5

Lorlatinib with or without chemotherapy in ALK-driven refractory/relapsed neuroblastoma: phase 1 trial results

In the format provided by the authors and unedited

Supplementary Table 1. Tumor Tissue Verses ctDNA Baseline ALK Results

Best overall response	Tumor *ALK*	Tumor source/ timepoint	ctDNA baseline	Prior ALK inhibitor
PD (Course 1)	F1174L	Bone/Relapse	F1174<br>F1245<br>R1275	Alectinib + Chemotherapy
PR (27 Courses)	D1276_R1279>E	Soft tissue/Relapse	D1276_R1279>E<br>G1202R	Crizotinib x 3 years
PD (Course 2)	ALK Amplification	Soft tissue/Refractory	ALK Amplification<br>F1174L	No Prior ALK TKI

Supplementary Table 2. MIBG Curie Scores at enrollment and Best Overall Response

Patient ID	32	10	30	12	9	26	40	17	3	11	21	25	2	38	31
**Enrollment Curie**	2	4	5	6	10	11	10	15	18	1	3	2	5	3	3
**Minimum Curie**	14	6	7	8	9	9	3	2	1	0	0	0	0	0	0
**Best overall response**	PD	PD	PD	PD	PD	PD	MR	MR	PR	SD	SD	SD	MR	PR	CR
**Curie Score Percent Change**	600	50	40	33.33	-10	-18.18	-70	-86.66	-94.44	-100	-100	-100	-100	-100	-100

PD; Progressive disease, SD; Stable disease, MR; Minimal response, PR; Partial response, CR; Complete response

Supplementary Table 3: Neuropsychological Administration Procedures by Age Group

<table>
<tr><th>Age at Testing (Years: Months)</th><th>Patient Testing/Self Report</th><th>Parent Report</th><th>Duration of Testing for Parents</th><th>Duration of Testing for Patients</th></tr>
<tr><td>1:1 to 1:11</td><td>1. Bayley-III (only at <u>baseline</u>, <u>course 2</u>, and <u>every 6 months</u> for duration of treatment- e.g., course 8, 14, etc.)</td><td>1. ABAS-3 Parent/Primary Caregiver Form (Ages 0-5)
2. PedsQL Infant Scales (13-24 months)</td><td>~ 20 minutes</td><td>~ 60 - 90 minutes</td></tr>
<tr><td>2:0 – 2:11</td><td>1. Bayley-III (only at <u>baseline</u>, <u>course 2</u>, and <u>every 6 months</u> for duration of treatment- e.g., course 8, 14, etc.)</td><td>1. ABAS-3 Parent/Primary Caregiver Form (Ages 0-5)
2. BASC-3 Parent Rating Scales-Preschool (Ages 2-5)
3. BRIEF- Preschool Version
4. PedsQL Generic Version – Parent Report for Toddlers (Ages 2-4)
5. PedsQL Multidimensional Fatigue Parent Report for Toddlers (Ages 2-4)</td><td rowspan="3">~ 50 minutes</td><td>~ 60 - 90 minutes</td></tr>
<tr><td>3:0 – 4:11</td><td>1. Cogstate (Detection, Identification)</td><td>1. ABAS-3 Parent/Primary Caregiver Form (Ages 0-5)
2. BASC-3 Parent Rating Scales-Preschool (Ages 2-5)
3. BRIEF- Preschool Version
4. PedsQL Generic Version – Parent Report for Toddlers (Ages 2-4)
5. PedsQL Multidimensional Fatigue – Parent Report for Toddlers (Ages 2-4)</td><td rowspan="2">~ 10 minutes</td></tr>
<tr><td>5:0 – 5:11</td><td>1. Cogstate (Detection, Identification)</td><td>1. ABAS-3 Parent/Primary Caregiver Form (Ages 0-5)
2. BASC-3 Parent Rating Scales-Preschool (Ages 2-5)
3. BRIEF- Preschool Version
4. PedsQL Generic Version – Parent Report for Young Children (Ages 5-7)
5. PedsQL Multidimensional Fatigue – Parent Report for Young Children (Ages 5-7)
6.</td></tr>
</table>

<table>
<tr>
<td>6:0 – 6:11</td>
<td>1. Cogstate
(Detection, Identification, One Card Learning, One Back, Groton Maze Learning Task)</td>
<td>1. ABAS-3 Parent Form (Ages 5-21)
2. BASC-3 Parent Rating Scales-Child (Ages 6-11)
3. BRIEF
4. PedsQL Generic Version – Parent Report for Young Children (Ages 5-7)
5. PedsQL Multidimensional Fatigue – Parent Report for Young Children (Ages 5-7)</td>
<td>~ 50 minutes</td>
<td rowspan="3">~ 30-40 minutes</td>
</tr>
<tr>
<td>7:0-7:11</td>
<td>1. Cogstate
(Detection, Identification, One Card Learning, One Back, Groton Maze Learning Task)</td>
<td>1. ABAS-3 Parent Form (Ages 5-21)
2. BASC-3 Parent Rating Scales-Child (Ages 6-11)
3. BRIEF
4. PedsQL Generic Version – Parent Report for Young Children (Ages 5-7)
5. PedsQL Multidimensional Fatigue – Parent Report for Young Children (Ages 5-7)
6. CSSRS – Children’s Version</td>
<td rowspan="3">~ 50 – 55 minutes</td>
</tr>
<tr>
<td>8:0-9:11</td>
<td>1. Cogstate
(Detection, Identification, One Card Learning, One Back, Groton Maze Learning Task)</td>
<td>1. ABAS-3 Parent Form (Ages 5-21)
2. BASC-3 Parent Rating Scales-Child (Ages 6-11)
3. BRIEF
4. PedsQL Generic Version – Parent Report for Children (Ages 8-12)
5. PedsQL Multidimensional Fatigue – Parent Report for Children (Ages 8-12)
6. CSSRS – Children’s Version</td>
</tr>
<tr>
<td>10:0-11:11</td>
<td>1. Cogstate
(International Shopping List, Detection, Identification, One Card Learning, One Back, Groton Maze Learning Task, International Shopping List Delayed)</td>
<td>1. ABAS-3 Parent Form (Ages 5-21)
2. BASC-3 Parent Rating Scales-Child (Ages 6-11)
3. BRIEF
4. PedsQL Generic Version – Parent Report for Children (Ages 8-12)
5. PedsQL Multidimensional Fatigue – Parent Report for Children (Ages 8-12)
6. CSSRS – Children’s Version</td>
<td>~ 35-45 minutes</td>
</tr>
<tr>
<td>12:0 – 12:11</td>
<td>1. Cogstate
(International Shopping List, Detection, Identification, One Card Learning, One Back, Groton</td>
<td>1. ABAS-3 Parent Form (Ages 5-21)</td>
<td></td>
<td></td>
</tr>
</table>

	Maze Learning Task, International Shopping List Delayed)	2. BASC-3 *Parent Ratings Scales – Adolescent (Ages 12-21)*<br>3. BRIEF<br>4. PedsQL Generic Version *– Parent Report for Children (Ages 8-12)*<br>5. PedsQL Multidimensional Fatigue *– Parent Report for Children (Ages 8-12)*<br>6. CSSRS	~ 50-55 minutes	~ 35-45 minutes
13:0 – 17:11	1. Cogstate *(International Shopping List, Detection, Identification, One Card Learning, One Back, Groton Maze Learning Task, International Shopping List Delayed)*	1. ABAS-3 *Parent Form (Ages 5-21)*<br>2. BASC-3 *Parent Ratings Scales – Adolescent (Ages 12-21)*<br>3. BRIEF<br>4. PedsQL Generic Version *– Parent Report for Teens (Ages 13-18)*<br>5. PedsQL Multidimensional Fatigue *– Parent Report for Teens (Ages 13-18)*<br>6. CSSRS		
18:0 – 25:11	1. Cogstate *(International Shopping List, Detection, Identification, One Card Learning, One Back, Groton Maze Learning Task, International Shopping List Delayed)*<br><br>2. ABAS-3 *Adult Self-Report Form (Ages 16-89)*<br>3. BASC-3 *Self-Report College (Ages 18-25)*<br>4. BRIEF - *Adult Self Report*<br>5. PedsQL Generic Version - *Young Adult Report (Ages 18-25)*<br>6. PedsQL Multidimensional Fatigue - *Young Adult Report (Ages 18-25)*<br>7. BDI-II<br>8. CSSRS	No Parent Reports	N/A	~80-90 minutes
≥26:0	1. Cogstate *(International Shopping List, Detection, Identification, One Card Learning, One Back, Groton Maze Learning Task, International Shopping List Delayed)*<br>2. ABAS-3 *Adult Self-Report Form (Ages 16-89)*<br>3. BRIEF - *Adult Self Report*<br>4. PedsQL Generic Version - *Adult Report (Ages* ≥26)<br>5. PedsQL Multidimensional Fatigue - *Adult Report (Ages* ≥26)<br>6. BDI-II<br>7. CSSRS			

Supplementary Figure 1. Responses on cohort A1 by age at enrollment. Statistically evaluated using two-sided T-Test.

Cohort	n	Mean Age (range) [n] for CR+PR+MR	Mean Age (range) [n] for SD+PD	t-test p-value
A1	23	10.5 (6-17) [7]	5.12 (2-9) [16]	0.0006

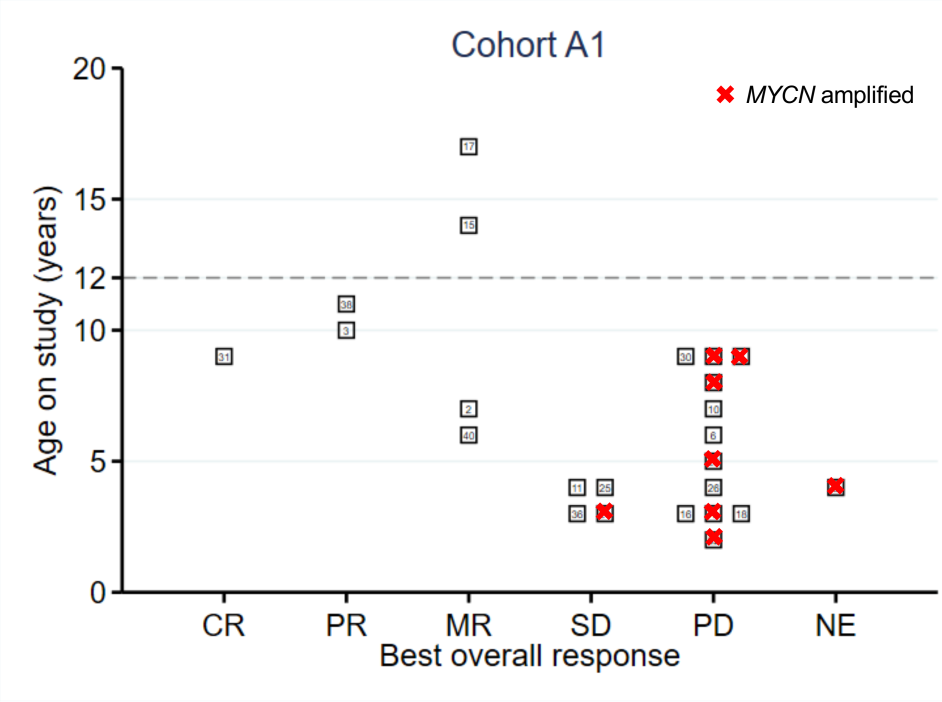

Supplement: Supplementary file 1 — Supplementary Tables 1–3 and Supplementary Fig. 1 [file 41591_2023_2297_MOESM1_ESM.pdf]
