# Supplementary material for: Lorlatinib with or without chemotherapy in ALK-driven refractory/relapsed neuroblastoma: phase 1 trial results
Source: Nat Med. 2023 Apr 3;29(5):1092–102. doi: 10.1038/s41591-023-02297-5 (PMC10202811; doi:10.1038/s41591-023-02297-5)
Supplement: Supplementary file 2 — Reporting Summary [file 41591_2023_2297_MOESM2_ESM.pdf]

nature portfolio

Corresponding author(s): Kelly Goldsmith

Last updated by author(s): Oct 18, 2022

Reporting Summary

Nature Portfolio wishes to improve the reproducibility of the work that we publish. This form provides structure for consistency and transparency in reporting. For further information on Nature Portfolio policies, see our Editorial Policies and the Editorial Policy Checklist.

Statistics

For all statistical analyses, confirm that the following items are present in the figure legend, table legend, main text, or Methods section.

n/a	Confirmed	
☐	☒	The exact sample size (*n*) for each experimental group/condition, given as a discrete number and unit of measurement
☒	☐	A statement on whether measurements were taken from distinct samples or whether the same sample was measured repeatedly
☐	☒	The statistical test(s) used AND whether they are one- or two-sided<br>*Only common tests should be described solely by name; describe more complex techniques in the Methods section.*
☒	☐	A description of all covariates tested
☒	☐	A description of any assumptions or corrections, such as tests of normality and adjustment for multiple comparisons
☐	☒	A full description of the statistical parameters including central tendency (e.g. means) or other basic estimates (e.g. regression coefficient) AND variation (e.g. standard deviation) or associated estimates of uncertainty (e.g. confidence intervals)
☐	☒	For null hypothesis testing, the test statistic (e.g. *F*, *t*, *r*) with confidence intervals, effect sizes, degrees of freedom and *P* value noted<br>*Give P values as exact values whenever suitable.*
☒	☐	For Bayesian analysis, information on the choice of priors and Markov chain Monte Carlo settings
☒	☐	For hierarchical and complex designs, identification of the appropriate level for tests and full reporting of outcomes
☒	☐	Estimates of effect sizes (e.g. Cohen's *d*, Pearson's *r*), indicating how they were calculated

Our web collection on statistics for biologists contains articles on many of the points above.

Software and code

Policy information about availability of computer code

Data collection	No software was used for data collection
Data analysis	SAS version 9.4. PK results were derived by noncompartmental analysis using Phoenix WinNonlin v8.3 and summarized by cohort and dose level and graphically presented using R v3.6.1

For manuscripts utilizing custom algorithms or software that are central to the research but not yet described in published literature, software must be made available to editors and reviewers. We strongly encourage code deposition in a community repository (e.g. GitHub). See the Nature Portfolio guidelines for submitting code & software for further information.

Data

Policy information about availability of data

All manuscripts must include a data availability statement. This statement should provide the following information, where applicable:

- Accession codes, unique identifiers, or web links for publicly available datasets
- A description of any restrictions on data availability
- For clinical datasets or third party data, please ensure that the statement adheres to our policy

The anonymized derived data from this study that underlie the results reported in this article will be made available, beginning 12 months and ending 5 years following this article publication, to investigators who sign a data access agreement and provide a methodologically sound proposal to medinfo@blueprintmedicines.com.

Human research participants

Policy information about studies involving human research participants and Sex and Gender in Research.

Reporting on sex and gender	Information on sex is obtained for all trial participants.<br>No information about gender was collected.<br>No analysis was performed based on sex.
Population characteristics	N/A no covariate analysis were conducted.<br>The patient demographics were described in full in the manuscript (Table 1). CONFIRM<br>Of all x enrolled patients, x were female and x were male. The median age was xxx range xxxx- should we add
Recruitment	Patients were recruited by participating investigators . The Investigator at each center ensured that the patients were given full and adequate oral and written information about the nature, purpose, possible risk, and benefit of the study. Patients were also notified that they were free to discontinue from the study at any time. Patients were given the opportunity to ask questions and allowed time to consider the information provided. All patients/legal guardian were provided written informed consent and an assent when applicable . Participants were not compensated. Inclusion and exclusion criteria are defined in full in tables in the manuscript Supplementary Information.<br>A total of 14 study centers were initiated, with patients enrolled at 13 of these centers, including 43 patients in the United States and 5 patients in Europe and 1 in Canada. Due to the geographical distribution of the study centers , participants may not represent the global general population. No other bias emerging from recruitment is expected.
Ethics oversight	The full protocol and all relevant documents were approved by the insititutional review board or independent ethics committee of each participating center and written informed consent and assent when applicable were obtained from all particpants. Each site's institutional review board approved the protocol and consent (Children's Hospital Los Angeles Institutional Review Board, CA, USA; Emory University Institutional Review Board, GA, USA; Children's Hospital of Philadelphia Institutional Review Board, PA, USA; Cincinnati Children's Hospital Institutional Review Board, OH, USA; Colorado Multiple Institutional Review Board, CO, USA; Cook Children's Health Care System Institutional Review Board, TX, USA; Comité de Protection des Personnes Ile-de-France X, Paris, France; Dana-Farber Cancer Institute, Office for Human Research Studies, MA, USA; London City & East Research Ethics Committee, Bristol, UK; Seattle Children's Institutional Review Board, WA, USA; SickKids Research Ethics Board, Toronto, CA; The University of Chicago Biological Sciences Division Institutional Review Board, IL, USA; University of California San Francisco Human Research Protection Program Institutional Review Board; University of Michigan Medical School Institutional Review Board, MI, USA). The trial was conducted in accordance with the Declaration of Helsinki, International Conference on Harmonization guidelines for Good Clinical Practice and local regulations.

Note that full information on the approval of the study protocol must also be provided in the manuscript.

Field-specific reporting

Please select the one below that is the best fit for your research. If you are not sure, read the appropriate sections before making your selection.

☒ Life sciences ☐ Behavioural & social sciences ☐ Ecological, evolutionary & environmental sciences

For a reference copy of the document with all sections, see nature.com/documents/nr-reporting-summary-flat.pdf

Life sciences study design

All studies must disclose on these points even when the disclosure is negative.

Sample size	Sample size for each of the three cohorts was determined by the standard 3+3 dose escalation design and dependent on the observed safety profile and evaluability of patients, with 5 dose levels for Cohort A, 2 dose levels for Cohort A2, 2 dose levels for Cohort B2.
Data exclusions	No data were excluded from the analyses. Analyses are presented for all enrolled patients as well as cohorts, which are defined in the manuscript.
Replication	This was a non randomized phase 1 clinical study to collect preliminary toxicity and response data. Replication of the results will be sought in the follow-up phase II and III studies.
Randomization	This was a non-randomized clinical trial. Data collection was prospective without controlling any covariates.
Blinding	This was an open label non-blinded trial as participants were not randomized and there was only one regimen for a given cohort.

Reporting for specific materials, systems and methods

We require information from authors about some types of materials, experimental systems and methods used in many studies. Here, indicate whether each material, system or method listed is relevant to your study. If you are not sure if a list item applies to your research, read the appropriate section before selecting a response.

Materials & experimental systems

n/a	Involved in the study
☒	☐ Antibodies
☒	☐ Eukaryotic cell lines
☒	☐ Palaeontology and archaeology
☒	☐ Animals and other organisms
☐	☒ Clinical data
☒	☐ Dual use research of concern

Methods

n/a	Involved in the study
☒	☐ ChIP-seq
☒	☐ Flow cytometry
☒	☐ MRI-based neuroimaging

Clinical data

Policy information about clinical studies

All manuscripts should comply with the ICMJE guidelines for publication of clinical research and a completed CONSORT checklist must be included with all submissions.

Clinical trial registration	NCT03107988.
Study protocol	PHASE 1 STUDY OF LORLATINIB (PF-06463922), AN ORAL SMALL MOLECULE INHIBITOR OF ALK/ROS1, FOR PATIENTS WITH ALK-DRIVEN RELAPSED OR REFRACTORY NEUROBLASTOMA. Clinical trial information can be found in clinicaltrials.gov/NCT03107988. The full protocol is not available due to contractual agreements.
Data collection	Patients were enrolled and data were collected from the 14 NANT institutions since the study was opened for accrual on September 1, 2017 until the data cut-off on September 13, 2022.
Outcomes	Primary outcome for this phase 1 study is the recommended phase 2 dose, which is defined as the dose level that has acceptable rate for dose limiting toxicity (DLT). DLT was summarized by cohort and dose level while toxicities were summarized by cohort and CTCAE grade. A second primary outcome is the pharmacokinetics (PK) of lorlatinib when combined with topotecan and cyclophosphamide in children. PK data, including AUCtau (area under plasma concentration-time curve/dosing interval) and Cmax (maximum plasma concentration), were summarized by cohort and dose level.<br><br>Anti-tumor response is the secondary outcome as defined by NANT response criteria version 2.0.
